# The impact of thyroid-stimulating hormone levels in euthyroid women on intrauterine insemination outcome

**DOI:** 10.1186/s12905-018-0541-0

**Published:** 2018-03-20

**Authors:** Gorkem Tuncay, Abdullah Karaer, Ebru İnci Coşkun, Demet Baloğlu, Ayşe Nihan Tecellioğlu

**Affiliations:** 1 Division of Reproductive Endocrinology and Infertility, Departments of Obstetrics and Gynecology, School of Medicine, Inonu University, 44315 Malatya, Turkey; 2 Departments of Obstetrics and Gynecology, School of Medicine, Inonu University, Malatya, Turkey

**Keywords:** Infertility, Intrauterine insemination, Thyroid, TSH

## Abstract

**Background:**

The aim of this study was to examine the effect of thyroid-stimulating hormone (TSH) levels on intrauterine insemination (IUI) outcomes among euthyroid women.

**Methods:**

A retrospective cohort study was conducted. A total of 302 women who started their first IUI cycle in our fertility center were included in this study. The patients were categorized into two groups based on their preconception TSH values: 0.38–2.49 mIU/Land 2.50–4.99 mIU/L. The clinical pregnancy rate was the main outcome parameter. As secondary parameters, we evaluated the differences in spontaneous abortion rate, live-birth delivery rate, and perinatal outcomes according to the preconception TSH threshold (< 2.5 and < 5.00 mIU/L).

**Results:**

There was no significant difference between the two groups with respect to clinical pregnancy, miscarriage, and live-birth rates with an odds ratio of 1.67 (95% CI: 0.79–3.53), 1.08 (95% CI: 0.09–13.1), and 1.79 (95% CI: 0.77–4.2), respectively. In addition, there were no significant differences in perinatal outcomes (gestation at delivery, birth weight, and neonatal intensive care unit–administration rate) between the two groups.

**Conclusions:**

Our findings indicate that among euthyroid patients, preconception TSH values in the high-normal range (between 2.5 and 4.9 mIU/L) do not have a negative effect on IUI outcomes.

**Trial registration:**

This study is retrospectively registered by Ethical Review Board at Inonu University in 19th December 2017; Ethics approval no is 2017–27-20.

## Background

At present, approximately 10–15% of couples seek medical assistance for infertility. The first documented application of artificial insemination in humans was done in the 1770s by John Hunter [1], and now, intrauterine insemination (IUI) is the first-line treatment in couples with unexplained infertility, cervical factors–related infertility, ejaculatory abnormalities, and male subfertility [2].

Thyroid disorders are one of the most common endocrine conditions affecting women during their reproductive age [3]. Thyroid disorders are associated with a number of adverse reproductive outcomes, including infertility and spontaneous abortion [4]. A growing body of literature addresses the implications of subclinical hypothyroidism on maternal and fetal health [5, 6], but few studies have evaluated the association between preconception thyroid-stimulating hormone (TSH) levels and pregnancy outcomes among euthyroid patients. Although the 2012 guidelines of the American Thyroid Association and the American Association of Clinical Endocrinologists recommend restricting the abnormality limit of serum TSH to 2.5 mIU/L even among euthyroid patients who plan to become pregnant [7], the 2017 guideline of the American Association Thyroid Association recommended restricting the abnormality upper reference limit of TSH to 4.0 mIU/L [8].

However, data regarding the optimal upper value of TSH and its effects on the outcome of fertility treatment remains controversial. Therefore, the present study was aimed at assessing the relative importance of TSH levels on the outcomes of women undergoing IUI.

## Methods

### Study population

**Fig. 1:**
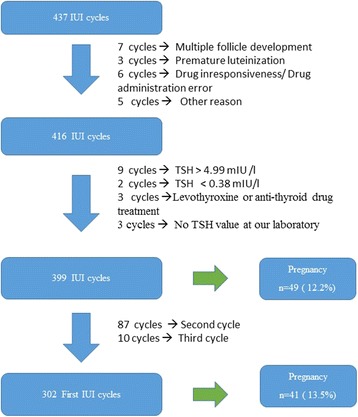
Flow chart of study

Data from IUI cycles that took place at the Division of Reproductive Endocrinology, Department of Obstetrics and Gynecology between April 1, 2014, and April 30, 2017, were retrospectively reviewed. A flow chart of this study is shown in Fig. 1. During this time, 437 IUI treatment cycles were initiated. Of these, 21 IUI treatment cycles were withheld due to polyfollicular development (7 cycles), premature luteinizing hormone (LH) surge (3 cycles), drug unresponsiveness/drug administration error (2 cycles), or for other reasons (9 cycles). Also, patients with incomplete TSH data (*n* = 3), thyroid dysfunction (a documented TSH value outside the normal range, i.e., TSH <  0.38 mIU/L or TSH > 5.00 mIU/L; 11 IUI treatment cycles), and patients under treatment with medications such as levothyroxine or anti-thyroid drugs (3 IUI treatment cycles) were excluded. Of the 399 remaining IUI cycles, 87 patients had undergone 2 IUI treatment cycles, and 10 patients had undergone 3 IUI treatment cycles. Finally, in the present retrospective cohort study, we analyzed the outcome of IUI in 302 women who started their first treatment cycle.

The clinical pregnancy rate (defined as the presence of a gestational sac and a fetal heartbeat through ultrasound) was taken as the primary outcome of this study. Secondary outcomes were implantation rate (defined as a standard serum assay for β-hCG showing levels above 10 IU/L), spontaneous abortion rate (defined as loss of a clinical pregnancy before 20 weeks), live-birth rate (defined as the live birth of a viable infant), and perinatal outcomes (gestation at delivery, birth weight, and neonatal intensive care unit–administration rate). We did not start any levothyroxine treatment in women with TSH value above 2.5 mIU/L after positive pregnancy result.

The study was approved by the Ethical Review Board at Inonu University (approval number: 2017–27-20) and was designed and carried out in accordance with the Declaration of Helsinki.

### Clinical and laboratory procedures

Because of randomized controlled studies have been shown that controlled ovarian stimulation in combination with IUI has been shown to result in significantly higher cumulative pregnancy rates per couple as compared to unstimulated intracervical insemination, controlled ovarian stimulation alone or IUI alone [9, 10], all the IUI cycles were combined with gonadotropic ovarian stimulation. The treatment was initiated on the second or third day of the cycle. These patients received mild ovarian hyperstimulation with recombinant follicle stimulating hormone (FSH) (Gonal F, Merck, Germany) or human menopausal gonadotropins (Menogon, Ferring, Switzerland). The initial prescribed dose of gonadotropin (37.5–75 IU per day) depended on the woman’s age, day-3 hormonal profile, and antral follicle count. Patients were monitored with the usual hormonal blood tests (estradiol and LH) and through vaginal ultrasound examination for follicle number, size, and endometrial maturation. Final follicular maturation was obtained by injection of 250 μg recombinant human chorionic gonadotropin (hCG) (Ovitrelle Merck, Germany) when leading follicle reached 18 mm in diameter. Insemination was performed 34–36 h after the hCG injection. In cycles with a spontaneous LH surge, IUI was performed 24 h after the surge.

After 48–72 h of abstinence and 2 h before the IUI, semen samples were collected at the laboratory. After an initial evaluation, discontinuous Percoll centrifugation with gradients of 80% and 40% was performed for sperm preparation as described in previous studies [11]. Semen samples were analyzed according to the 2010 World Health Organization criteria [12]. Fresh husband’s sperm with a soft catheter (Laboratoire C.C.D., Paris, France) were used for the insemination process.

All patients undergoing fertility treatments in our center are routinely screened for thyroid disease and TSH values was determined before initiating ovarian stimulation. To avoid any inter-laboratory bias, only patients who performed their laboratory screening in our hospital were included. Serum TSH was assessed by using an electrochemiluminescent immunoassay with a Beckman Coulter immunoassay analyzer (USA). All examined serum parameters were determined in the ISO-certified central laboratory. The reference values were 0.38–5.00 mIU/L for TSH. The inter- and intra-assay coefficient of variation percentages (% CV) were 6.0 and 4.2 for TSH, respectively.

### Statistical analysis

Data were stored and analyzed by using the Statistical Package for Social Science Software for Windows, release 11.0 (SPSS, Inc; Chicago, IL, USA). A power analysis was performed with an alpha of 0.05. A sample size of 110 (55 per group) was required to detect a 5% difference regarding to success of IUI between groups. The normality of distribution was assessed using the Kolmogorov-Smirnov test. Variables with a normal distribution were presented as mean ± standard deviation (SD), and variables with a skewed distribution were presented as median and interquartile range (IQR). Patients were split into two groups defined by TSH values of 0.38–2.49 mIU/l and 2.5–4.99 mIU/Ll, respectively. Groups were compared using the Student’s t-test for parametric data and the Mann-Whitney U test for non-parametric data. The chi-square or Fisher’s exact tests were used for categorical variables. Odds ratios (OR) are given, including a 95% confidence interval (95% CI). In multiple logistic regression analysis, the clinical pregnancy rate was designated as a dependent variable and the following parameters were analyzed as possible influencing factors on the outcome of IUI: female age, BMI (defined as weight (kg) /[height (m^2^)], number of births, TPMS (defined as semen volume x motile sperm count x sperm motility), ovarian reserve (day-3 antral follicle count), and TSH. A *p*-value less than 0.05 was considered statistically significant.

## Results

A total of 302 women who underwent their first IUI cycles were included in the analysis. Of these, 233 women (77.2%) had TSH values between 0.38–2.49 mIU/L (the low-TSH group), and 69 women (22.8%) had TSH values between 2.50–5.00 mIU/L (the high-TSH group). The clinical characteristics and smoking habits of the study participants are outlined in Table 1. There was no significant difference in both female and partner mean age between the two groups (the mean female age was 29.3 ± 4.4 years in the low-TSH group vs. 29.1 ± 4.4 years in the high-TSH group with *p* = 0.76, and mean male age was 32.8 ± 4.3 years in the low-TSH group vs. 33.4 ± 4.2 years in the high-TSH group with *p* = 0.31). The mean BMI was 25.1 ± 3.9 in women in the low-TSH group and 25.7 ± 4.0 in the high-TSH group (*p* = 0.36). The mean duration of infertility and number of births were comparable between the two groups. A total of 29/233 (12.4%) women in the low-TSH group smoked cigarettes, whereas only 5/69 (7.2%) women smoked in the high-TSH group. Although there was higher proportion of women in low-TSH group smoke cigarettes than women in high-TSH group, there was no statistically significant difference (*p* = 0.23). Moreover, ovarian reserves in terms of day-3 FSH levels and antral follicle count were comparable between two groups. Likewise, there was no statistically significant difference regarding sperm-quality parameters between the two groups (Table 1).

**Table 1:** Characteristics of study population of women undergoing IUI cycles according to TSH level (mIU/l)^a^

Variables	TSH 0.40–2.49 mIU/l	TSH 2.50–4.99 mIU/l	*p-*values
	*n* = 233	*n* = 69)	
TSH level (mIU/l)	1.44 ± 0.53	3.29 ± 0.63	< 0.001
Age			
Female age (y)	29.3 ± 4.4	29.1 ± 4.4	0.76
Partner age (y)	32.8 ± 4.3	33.3 ± 4.2	0.31
Medical history			
Duration of marriage (y)	4 (3–7)	4 (3–6)	0.98
Duration of infertility (y)	3 (2–4.5)	3 (2–5)	0.39
Parity			0.92
0	197 (84.5%)	59 (85.6%)	
≥ 1	36 (15.5%)	10 (14.5%)	
Lifestyle factors			
BMI (kg/m^2^)	25.1 ± 3.9	25.7 ± 4.0	0.36
No. Smokers (%)	204 (87.6%)	64 (92.8%)	0.23
29 (12.4%)	5 (7.2%)		
Ovarian reserve			
Day 3 FSH (U/l)	6.2 ± 2.1	6.7 ± 1.9	0.36
Day 3 E2	56.1 ± 37	53.5 ± 30	0.54
AFC	14 (8–20)	13 (9–20)	0.82
Sperm quality			
Sperm conc. (×10^6^)	47.5 (30–62.5)	41 (26–58)	0.11
Sperm motility	50 (45–53.5)	50 (45–52)	0.13
TPMS (×10^6^)	42.3 (24–67.3)	31.2 (18.2–59.2)	0.06
Sperm morphology	2(1–3)	3(2–3)	0.32

^a^At first cycle according to TSH level

Data shown as means ± standard deviations or median (IQR)

There were no significant difference between the groups in respect to the median total gonadotropin dose used (562.5 IU in the low-TSH group vs. 581 IU in the high-TSH group [*p* = 0.66]). There were also no significant differences between the two groups for the median day of hCG trigger (9 days for both groups [*p* = 0.83]), the median number of pre-ovulatory follicles (follicles ≥14 mm; 1 follicle for both groups [*p* = 0.78]), or the mean endometrial lining thickness (9.5 ± 1.7 mm in the low-TSH group vs. 9.7 ± 2.4 mm in the high-TSH group [*p* = 0.62]) (Table 2).

**Table 2:** Associations of TSH level with treatments parameters of IUI

Variables	TSH 0.40–2.49 mIU/l	TSH 2.50–4.99	*p-*value
	(*n* = 233)	(*n* = 69)	
Total gonadotropin dose (U/ml)	562.5 (406–862.5)	581 (403–1050)	0.66
Day of hCG trigger	9 (7–12)	9 (7–14)	0.83
Endometrial thickness (mm)	9.5 ± 1.7	9.7 ± 2.4	0.62
Number of pre-ovulatory follicles (14 mm)	1 (1–2)	1 (1–2)	0.78

Data shown as means ± standard deviations or median (IQR)

The overall pregnancy rate was 41/302 (13.57%). After the exclusion of 3 losses in the biochemical phase of pregnancy, the clinical pregnancy rate was 38/302 (12.5%). The clinical pregnancy rate was similar between two groups. Twenty-eight (28) patients (12%) out of the 233 patients in the low-TSH group became pregnant, whereas the clinical pregnancy rate was 13/69 (18%) in the high-TSH group (*p* = 0.17) (Table 3). Of the 41 clinical pregnancies, there were no multiple pregnancies.

**Table 3:** Associations of TSH level with outcomes of IUI

Variables	TSH 0.40–2.49 mIU/L	TSH 2.50–4.99	*p-*value	OR (95% CI)
	(*n* = 233)	(*n* = 69)		
Implantation rate	28	13	0.14	1.7 (0.83–3.49)
Biochemical pregnancy loss	2	1	1.00	1.1 (0.09–13.1)
Clinical pregnancy	26	12	0.17	1.67 (0.79–3.53)

The obstetrics outcome were similar between the two groups. There was no significant difference in the preterm birth rate, gestational age at birth, birth weight, and caesarean section ratio between the two groups (Table 4).

**Table 4:** Associations of TSH level with obstetric outcomes of IUI

Variables	TSH 0.40–2.49 mIU/L	TSH 2.50–4.99	*p-*value
	(*n* = 26)	(*n* = 12)	
Intrauterine fetal death	2 (7.7%)	0	1.00
Preterm birth	2 (7.7%)	1 (8.3%)	1.00
Term birth	22 (84.6%)	11 (91.7%)	0.55
Duration of gestation (d)	263.83 ± 15.8	266.6 ± 19.6	0.66
C/S section	15/24 (62.5%)	6 /12 (50%)	0.47
NICU administration	2 (7.7%)	2 (16.6%)	0.57
Birth weight(g)	2923 ± 728	3102 ± 628	0.47

d: day; g: gram; NICU: neonatal intensive care unit administration rate

After adjusting for female age, BMI, parity, TPMS, ovarian reserve (day-3 antral follicle count) and low- and high-TSH groups, there was no association between any parameters and IUI success rate (for age, adjusted OR (AOR): 0.98 [95% CI: 0.90–1.07], *p* = 0.69; for BMI, AOR: 0.92 [95% CI: 0.78–1.12] for parity, AOR: 1.5 [0.73–3.3], *p* = 0.25; for antral follicle count (AFC) AOR: 0.97 [95% CI: 0.92–1.03], *p* = 0.37; for TPMS, AOR: 1.00 [0.99–1.01]; for TSH groups, AOR: 0.5 [0.23–1.07], *p* = 0.07).

## Discussion

In this cohort study, women with preconception TSH values between 2.5 and 4.99 mIU/L had similar fertility outcomes to women with TSH values < 2.5 mIU/L. The clinical pregnancy rate was similar between the two groups. Twenty-eight (28) patients (12%) out of the 233 patients in the low-TSH group became pregnant, whereas the clinical pregnancy rate was 13/69 (18%) in the high-TSH group.

The strengths of this study include its unique population of women receiving infertility treatment with IUI. In addition to, there was no statistically significant difference regarding to sperm quality parameters. Sperm concentrations, motility, and morphology are similar between two groups. Although TPMS count is better in men who’s partner in low TSH group than men’s whose partners in high TSH group (42.3 × 10^6^ and 31.2 × 10^6^, respectively), both TPMS levels are above than 10 × 10 ^6^, which is an important threshold for success of IUI [13]. Therefore, this difference are not both statistical and clinical significance. All patients in our fertility clinics routinely undergo TSH measurement, and all have outcomes meticulously documented. To avoid any inter-laboratory bias, only patients who performed their laboratory screening in our hospital were included. The majority of the characteristics of both groups were similar, thereby excluding confounding the outcome of the IUI by obstetrical history, lifestyle factor, ovarian reserve, or sperm quality. Despite these advantages, using a population of infertile women undergoing IUI has some limitations. It is not known whether these findings are generalizable to women without known fertility problems or women undergoing infertility treatment with assisted reproductive technologies. Another limitation is that the thyroid-antibody status and free-T4 levels of these women was not known.

Our results are in agreement with those recently reported in studies by Reh et al. [14] and Karmon et al. [15]. These authors observed no significant differences in clinical pregnancy rates among women with TSH levels of 0.4–2.4 mIU/L and those with levels > 2.5 mIU/L. Consistent with this study, Reh et al. [14] did not see any difference in miscarriages rates among women in low- and high-TSH groups. On the contrary, Karmon et al. [15] found that preconception TSH levels were inversely related to spontaneous abortion and positively related to live birth among women who achieved a clinical pregnancy.

The role of TSH on pregnancy outcomes in the case of IUI is widely debated. Initial studies of women in the Europe and United States led to recommendations for TSH upper reference limit of 2.5 mIU/L in the first trimester and 3.0 mIU/L in the second and third trimester [16, 17]. According to the 2012 guidelines of the American Association of Clinical Endocrinologists as well as the American Thyroid Association, the preconception and first trimester–pregnancy thresholds for the upper limit of TSH should ideally be > 2.5 mIU/L to diagnose and treat subclinical hypothyroidism in women attempting pregnancy [7]. This recommendation reflects the understanding that physiologically, hCG cross-reacts with TSH receptors, resulting in a decline in TSH levels [18].

However more, recent studies in pregnant women in Asia (China, Korea and India) have demonstrated that only a modest reduction in upper reference limit [19–21]. According this results, in the recent guidelines of American Thyroid Association, it is stated that the lower reference range of TSH can be reduced by approximately 0.4 mIU/L while the upper reference range is reduced by approximately 0.5 mIU/L and for the patients in first trimester, this corresponds to a TSH upper limit of 4.0 mIU/L [8].

In a recent guidelines of the Practice Committee of the American Society for Reproductive Medicine, it is stated that there is insufficient data to show that TSH levels between 2.5 and 4 mIU/l are associated with miscarriage and adverse pregnancy outcomes [22]. In addition, one has to keep in mind the less obvious but potential hazards that may be associated with possible overtreatment with levothyroxine. A recent study by Korevaar et al. [23] found that maternal thyroid excessive treatment in early childhood was associated with adverse effects childhood IQ and brain morphology.

## Conclusion

In conclusion, the present study does not suggest that preconception TSH values > 2.5 mIU/L among euthyroid women undergoing IUI are associated with adverse outcomes. The present study demonstrated that a stricter TSH cut-off in the preconception period may not improve a euthyroid woman’s ability to either conceive following treatments or carry a pregnancy to term. Future studies should separate and clarify the potential benefits of treating asymptomatic women with a high-normal TSH level who are planning to become pregnant or who are already pregnant.

## Abbreviations

FSH: Follicle stimulating hormone
hCG: Human chorionic gonadotropin
IUI: Intrauterine insemination
TSH: Thyroid-stimulating hormone
